# The oral-gut microbiome axis in breast cancer: from basic research to therapeutic applications

**DOI:** 10.3389/fcimb.2024.1413266

**Published:** 2024-11-21

**Authors:** Lan Huang, Chun Jiang, Meina Yan, Weimin Wan, Shuxiang Li, Ze Xiang, Jian Wu

**Affiliations:** ^1^ Department of Clinical Laboratory, The Affiliated Suzhou Hospital of Nanjing Medical University, Suzhou Municipal Hospital, Gusu School, Nanjing Medical University, Suzhou, Jiangsu, China; ^2^ Zhejiang University School of Medicine, Hangzhou, Zhejiang, China

**Keywords:** oral-gut microbiome axis, gut microbiota, oral microbiota, BC, tumor microecology

## Abstract

As a complicated and heterogeneous condition, breast cancer (BC) has posed a tremendous public health challenge across the world. Recent studies have uncovered the crucial effect of human microbiota on various perspectives of health and disease, which include cancer. The oral-gut microbiome axis, particularly, have been implicated in the occurrence and development of colorectal cancer through their intricate interactions with host immune system and modulation of systemic inflammation. However, the research concerning the impact of oral-gut microbiome axis on BC remains scarce. This study focused on comprehensively reviewing and summarizing the latest ideas about the potential bidirectional relation of the gut with oral microbiota in BC, emphasizing their potential impact on tumorigenesis, treatment response, and overall patient outcomes. This review can reveal the prospect of tumor microecology and propose a novel viewpoint that the oral-gut microbiome axis can be a breakthrough point in future BC studies.

## 1 Introduction

Breast cancer (BC), the complicated and heterogeneous disorder, influences millions of women worldwide. It occupies 1/4 of female caners and exhibits the highest morbidity among female cancers, especially in transitioning countries (Arnold et al., 2022; Heer et al., 2020). Consistent with GLOBOCAN 2022 analysis of cancer patients in either developed countries or developing countries, major concerned cancers including BC, may increase eventually (Fatima et al., 2023; Wang et al., 2019, Wang et al., 2024; Liu et al., 2024). The World Health Organization (WHO) has initiated the Global BC Initiative to enhance awareness about women’s health issues and improve public health interventions (Anderson et al., 2021). Numerous studies have been performed to understand underlying mechanisms, risk factors, diagnostic techniques, and treatment options for BC (Koual et al., 2020; Zhang et al., 2023c). A large and growing body of literature has concentrated on the drug resistance, pharmacological anti-tumor efficiency, as well as clinicopathological and prognostic significances of BC, especially the triple-negative breast cancer (TNBC) (Geng et al., 2023; Zhang et al., 2023a; Kwon et al., 2023; Chen et al., 2023). Actually, early BC screening and prevention are vital for selecting treatment strategies for BC.

Recently, an increasing number of studies are conducted on different microbial species in the human body, their biological characteristics, and their correlations with various diseases (Wu et al., 2022b; Xiang et al., 2023). This growth can be attributed to the rapid advancements in microbiology and bioinformatics, as well as the improvements in microbial profiling technologies. In addition, it is becoming evident that every microbial habitat possesses unique microbial populations with different impacts on physiological homeostasis (Liu et al., 2022; Sun et al., 2023). Research has consistently indicated the association between dysbiosis in the microbiota and tumor initiation. Specific microbial species are correlated with a higher or lower risk of various cancers. For instance, primary gastric lymphoma carries a heightened susceptibility to gastric cancer or precancerous lesions, with the risk potentially related to *Helicobacter pylori* infection (Feng et al., 2023; Zhang et al., 2023e), while certain strains of *Escherichia coli* have been related to colon cancer (Blanco-Miguez et al., 2023; Wirbel et al., 2019). Emerging evidence indicates that breast microbiota is related to BC. Dysbiosis in the mammary gland and axillary region is associated with a higher BC risk. Specific bacterial species, including *Staphylococcus* and *Streptococcus*, have been found to be more abundant among BC patients than normal controls (Urbaniak et al., 2016; Kost et al., 2023). These microbes may promote chronic inflammation, DNA damage, and immune system dysregulation, therefore facilitating tumorigenesis. More investigations are warranted to completely explore the mechanisms and clinical implications of microbiota in different cancer.

The concept of multiple organ microbiota axes has entered the public awareness, including the ‘skin-gut’ microbiome axis, the ‘microbiota-gut-brain’ axis, the ‘lung−gut’ microbial axis, and the ‘oral-gut-liver’ axis (Russo et al., 2023a; Molina-Mateo et al., 2023; Nuzum et al., 2023; Wei et al., 2023; Niu et al., 2023). Recent research indicates that the construction of the microbiota-metabolic axis, such as the “gut microbiota-metabolites-serum lipid index” axis, contributes to identifying alternative strategies to improve high-fat diet (HFD)-induced hyperlipidemia (Zeng et al., 2023a). Based on these studies, it can be inferred that each microorganism exhibits different microbial population patterns in different habitats. The interplay and interactions between physical and biological communities among different organs influence the genesis and progression of various disorders.

Gut and oral microbiomes are two main microbial ecosystems in human body, which are vital for microbiome−related disorders. The most common bacteria in the oral cavity are members of *Firmicutes*, *Proteobacteria*, and *Actinobacteria*. However, compared to the gut microbiota, which undergoes more significant fluctuations, the oral microbiota ecosystem exhibits a relatively stable nature. The gut microbiota is a diverse and intricate microbial ecosystem that primarily comprises bacteria, viruses, fungi, and archaea. They work in coordination with the human body to provide a range of health benefits, such as enhancing the intestinal barrier, supporting the immune system, and providing energy (Schwabe and Jobin, 2013). The oral-gut microbiome axis indicates the dynamic relationship of microbial communities within oral cavity and the gut, and it is of great importance for human health and disease. Recent studies have emphasized the influence of oral-gut microbiome axis on cancer development and progression (Liu et al., 2022; Russo et al., 2023b; Elghannam et al., 2023; Jeong et al., 2023). The oral-gut microbiome axis can impact cancer through multiple mechanisms. Microbes can directly interact with host cells, affecting immune responses, inflammation, and DNA damage, which are key factors in cancer development. Evidence from mechanistic studies reveals that the microbiota can interact with host immune cells and epithelia by releasing a wide array of metabolites, proteins, and macromolecules, further affecting tumor cell behavior, angiogenesis, and treatment response. The oral-gut microbiome axis also regulates systemic metabolism, endocrine signaling, and drug metabolism, therefore influencing cancer progression and treatment outcomes (Pushalkar et al., 2018; Routy et al., 2018; Li et al., 2022; Qu et al., 2023; Wong and Yu, 2023).

Currently, the oral-gut microbiome axis exhibits a closer correlation with intestinal disorders due to the intricate relationship between microbial communities and gut health. The oral-gut microbiome axis emerges as the significant research topic in colorectal cancer (Liu et al., 2022; Lam et al., 2022). However, studies on the correlation of microbial communities of oral-gut microbiome axis with another major female cancer, BC, have certain limitations. This study provides the comprehensive review on the research related to remicrobial communities in the oral-gut microbiome axis in relation to BC. It is vital to understand the impact of oral-gut microbiome axis on BC, which holds implications for clinical practice. Oral-gut microbiome may be a source of biomarkers for BC risk assessment, early detection, and monitoring of treatment response.

## 2 The oral-gut microbiome axis

### 2.1 The oral and gut

The oral cavity and the gut are integral components in human body, serving vital functions. Anatomically, the human digestive system comprises the digestive tract and accessory digestive organs, including the liver and pancreas. The digestive tract spans from oral cavity to the gut, specifically the anus, making oral cavity and gut the anatomically neighboring regions that are interconnected through the gastrointestinal tract (Schmidt et al., 2019; Liu et al., 2022). Oral cavity is the primary site in the digestive tract, encompasses structures such as teeth, gums, and the tongue, and it fulfills the critical roles of mechanically breaking down food by chewing and initiating the digestive process. Furthermore, it hosts a complex microbial community that influences oral health and is important for systemic health (Liu et al., 2022; Eltay and Van Dyke, 2023). Especially, it provides various binding sites that microorganisms can adhere to and colonize. The gut, specifically the intestines, is primarily responsible for nutrient digestion and absorption (Belkaid and Hand, 2014). In the gut resides, there are various microorganisms called the gut microbiota. This intricate microbial community is important for maintaining overall health. It actively participates in digestion, synthesizes essential vitamins, modulates immune system, and contributes to defense against harmful pathogens (Tilg et al., 2020; Zeng et al., 2023b). Therefore, there is a potential correlation of oral microbes with the initiation and progression of gastrointestinal diseases (Liu et al., 2022). In fact, the oral and gut microbiomes exert a vital impact on keeping homeostasis in human body. The presence of oral-gut barrier ensures a clear separation in microbial distribution between oral cavity and gastrointestinal tract. Many studies have reported that certain bacteria, when colonizing specific sites and causing dysbiosis of oral and gut microbiota, may trigger the genesis and progression of various tumors, while also exerting a series of impacts on the surrounding microenvironment (**Table 1**).

**Table 1:** Dysbiosis of oral-gut microbiomes in various cancers in recent years.

Cancers	Dysbiosis of Oral-gut microbiomes	Impact	Reference
Gastric cancer	*Helicobacter pylori* cause dysbiosis of the oral-gut axis microbiota; Lactic acid bacteria and the oral microflora are frequently shown to be enriched. *Firmicutes*, *Actinobacteria*, *Peptostreptococcus stomatis*, *Johnsonella ignava*, *Neisseria mucosa* and *Prevotella pleuritidis* were enriched.	Exerting an influence on the occurrence and progression. Inducing chronic inflammation or facilitating the generation of nitroso compounds.	(Chen et al., 2022b; Yang, 2023; Wu et al., 2022a; Yang et al., 2022; Asili et al., 2023)
Colorectal cancer	Oral microbiomes and oral biofilm-associated bacteria such as *Spirochaetota* phylum, *Spirochaetia* classes, *Gracilibacteria* order, *Absconditabacteriales*, *Fusobacterium*, *Gemella*, *Parvimonas, Granulicatella*, *Leptotrichia*, *Peptostreptococcus*, *Campylobacter*, *Selenomonas*, *Porphyromonas*, and *Prevotella* were enriched. Gut microbiomes: *Peptostreptoccales* and *Tissierellales*; a decrease in the *Pseudomonadaceae* and *Yersiniaceae* increased.	Having an impact on CRC occurrence and AP progression in CRC.	(Senthakumaran et al., 2023; Russo et al., 2023b; Kato et al., 2023; Boyanova et al., 2023; Guo et al., 2018; Zhang et al., 2020b)
Esophageal cancer	*Tannerella forsythia* and *Porphyromonas gingivalis* were related to a higher risk of esophageal adenocarcinoma and esophageal squamous cell carcinoma. The *Streptococcus* number significantly increased, while *Faecalibacterium* number evidently decreased.	Promoting cancer genesis and progression and impacting cancer patient prognosis.	(Kong et al., 2023; Peters et al., 2017; Wang et al., 2021; Munteanu et al., 2023; Hasuda et al., 2023)
Prostate cancer	The abundance of *Porphyromonas gingivalis* and *Bacteroides massiliensis* has been positively correlated with the incidence of cancer. *Treoponema denticola* was observed in prostatic secretion samples which suggested potential bacterial movement from oral cavity to prostate. Oral bacteria, *Fusobacterium nucleatum* and *Granulicatella adiacens* were detected in cyst fluid from individuals with high-grade dysplasia which was correlated with intraductal papillary mucinous neoplasm (IPMN)	Establishing a proinflammatory or anticancer milieu by modulating host physiology.	(Wang et al., 2021; Munteanu et al., 2023; Javier-Desloges et al., 2022; Ohadian Moghadam and Momeni, 2021; Porter et al., 2018; Tan et al., 2023; Gaiser et al., 2019)
Pancreatic cancer	*Fusobacterium nucleatum* has been strongly associated with cancer and it has been identified as a common bacterium; *Pseudomonas* has been enriched in patients with cancer.	Influencing tumor development and treatment through the activation of oncogene pathway, promoting oncogene metabolic pathways, changing tumor cell growth, and promoting chronic inflammation for inhibiting tumor immunity.	(Bastos et al., 2023; Chai et al., 2023)

### 2.2 Bidirectional microbial translocation between oral and gut microbiota

Recently, numerous studies have revealed the presence of microbial translocation between oral and gut microbiota. In general, intestinal microbial colonization in the oral cavity is uncommon. However, specific conditions such as poor hygiene or immunological alterations may facilitate microorganisms translocation from gastrointestinal tract to oral cavity (Liu et al., 2022). *Bifidobacterium*, a bacterial genus, is known to be the predominant microorganism present in the intestinal tract of newborns. However, recent investigations have identified gut-dwelling *Bifidobacterium* in oral fluid in neonates (Makino, 2018). In individuals with inflammatory bowel disease (IBD), gut microbes have the capacity of impacting oral flora composition in a direct or indirect manner by modulating the host’s immune function (Liu et al., 2022). In addition to *in vivo* transmission, gastrointestinal microorganisms such as *Helicobacter pylori*, *Vibrio cholerae* or enteric viruses like Hepatitis A virus and Hepatitis E virus, can be transmitted through fecal-oral axis by directly contacting or indirectly contacting with polluted fluids and food (De Graaf et al., 2017; Bui et al., 2016; Chapman et al., 2023). Recent research has revealed that *Klebsiella pneumoniae* has the ability to disseminate to sterile sites in one host or transmit to another host via the fecal-oral route (Hudson et al., 2022).

Under specific circumstances, oral microbes have the potential to overcome physicochemical barriers existing between oral cavity and the gut, allowing for their potential translocation into the gut (Park et al., 2021). The changes observed in gut microbiome are caused by a decrease in stomach acidity, which enables a higher survival rate for bacteria ingested along with food and oral mucus. Therefore, the species present in the oral microbiome exhibit increased abundance in the gut microbiome of individuals who use proton pump inhibitors (PPIs) (Imhann et al., 2016). As suggested by Koji Atarashi, certain *Klebsiella* bacterial strains present in the salivary microbiome can colonize in the gut (Atarashi et al., 2017). Another research has shown that the prevalence of oral bacterial transition such as *Porphyromonas*, *Fusobacterium*, and *Pseudoramibacter* to gut increases among the elderly relative to adults, hoping that oral health care among the elderly can influence the gut microbiota composition (Iwauchi et al., 2019). In patients diagnosed with inflammatory bowel disease (IBD), *Haemophilus* and *Veillonella* abundances are significantly enriched within gut mucosa. These particular microbial species are recognized as oral commensal microbes (Gevers et al., 2014).

In tumor-related diseases, several studies have reported that significant dysbiosis is observed in the colonic environment of tumor-bearing colons, indicating a considerable degree of microbial imbalance. *Gemella*, *Peptostreptococcus*, *Parvimonas*, and other oral-origin microbes form a robust symbiotic network that characterizes the metacommunity associated with colorectal cancer (CRC). Through probabilistic partitioning of relative abundance profiles, the CRC-associated metacommunity is primarily dominated by members of oral microbiome (Nakatsu et al., 2015). According to the above results, under circumstances of disrupted mucosal homeostasis, the normal human oral microbiota possesses the ability of invading and colonizing gut mucosa, potentially transforming into an opportunistic pathogen (Park et al., 2021). In an observational study, *Streptococcus salivarius* and *Streptococcus parasanguinis* had significantly increased total relative abundances in the intestinal tract of elderly participants or those with dental plaque accumulation. This study provides evidence for oral bacterial translocation into the gut (Kageyama et al., 2023).

Among the gut-colonized oral bacteria, Hongze Zhang et al. reported that *Fusobacterium* abundance significantly increased, while *Prevotella* and *Ruminococcus* abundances obviously reduced among CRC samples (Zhang et al., 2023b). Oral strains of *Fusobacterium nucleatum* could be usually detected in CRC tissues, indicating the possible oral origin of *F. nucleatum* in cancers. In addition, the *F. nucleatum* level is related to the development, metastasis, and resistance to chemotherapy in colorectal cancer, potentially through the induction of autophagy pathways (Boyanova et al., 2023; Fujiwara et al., 2020; Sun et al., 2020). Certain oral anaerobes, such as *Fusobacterium necrophorum* and *Fusobacterium nucleatum*, are components of normal pharyngeal microbiota. Their potential translocation to other gastrointestinal tissues has been related to carcinogenesis (Boyanova et al., 2023). In conclusion, there is a significant intrinsic connection between oral and gut microbiota. Bidirectional microbial translocation between the oral and gut can mutually reshape microbial ecosystems of both environments, finally modulating the physiopathological processes of gastrointestinal diseases, including cancer. Investigating the association of oral-gut microbiota with various diseases may be vital for disease prediction and future therapeutic approaches.

## 3 Oral and gut microbes in breast cancer

Human commensal microbiota is vital for both health and disease. The microbial composition within various organs of human body reflects the differences in host genetics and lifestyle. The dynamic equilibrium between microbiota and host makes local and systemic effects on the body, and microbial dysbiosis may increase a variety of health risks. Many bacteria can encode genes for estrogen-metabolizing enzymes, therefore influencing the regulation of serum estrogen contents. On the contrary, estrogen-like compounds can enhance the growth and development of some bacteria. Therefore, the interaction of the microbiota with endogenous hormones, and estrogen-like compounds, may make synergistic effects in providing protection against diseases but also potentially increasing the hormone-related disorder risk. Recently, microbiota in BC patients has been found to be different from normal females, implying that some bacteria are related to the occurrence of BC and diverse cancer treatment responses. The gastrointestinal tract harbors the highest abundance of commensal microbiota, while oral microbiota, at the entry point of human digestive tract, consists of nearly 700 different microbial species, second only to the gut microbiota in diversity. Changes in the abundance of these two most abundant microbial habitats have also been correlated with BC (**Table 2**).

**Table 2:** Alterations in the abundance of oral and gut microbiota in BC.

Microbiome types	Microbiota sources	Increase (↑) or decrease (↓) in abundance	Reference
Oral microbes	Bacteria isolated from breast tissue of Caucasian women.	*Proteobacteria*, *Firmicutes* phyla, *Fusobacteria* and *Streptococcus* genera↑	(Urbaniak et al., 2014; Hao et al., 2019)
	Breast tissue among BC patients.	*Proteobacteria*, *Fusobacterium nucleatum* ↑	(Saud Hussein et al., 2021; Wade, 2013)
	BC in Ghana.	*Porphyromonas*, *Fusobacterium* ↓	(Wu et al., 2022c)
	BC patients undergoing chemotherapy.	*Neisseria*↓, *Escherichia*/*Shigella*↑	(Klymiuk et al., 2022)
Gut microbes	Newly diagnosed BC patients.	*Firmicutes*↑, *Bacteroidetes*↓ *Odoribacter sp*, *Butyricimonas sp* and *Coprococcus sp*, ↓	(Jaye et al., 2021; Bobin-Dubigeon et al., 2021)
	Patients with BC.	*Clostridiales*, *Bacillus*, *Staphylococcus*, *Enterobacteriaceae*, *Comamondaceae*, *Bacteroidetes*, *Porphyromonas* and *Peptoniphilus*↑ *Faecalibacterium*↓	(Saud Hussein et al., 2021; Urbaniak et al., 2016; Haque et al., 2022; Wang et al., 2022; Ma et al., 2022; Mikó et al., 2018; Ruo et al., 2021; Ma et al., 2020)
	Postmenopausal BC patients	*Escherichia coli*, *Enterococcus gallinarum*, *Actinomyces*, *Shewanella putrefaciens*, and *Erwinia amylovora*↑ *Eubacterium eligens*, *Lactoisacus*, *Pediococcus* and *Desulfovibrio*↓	(Barrea et al., 2023; Zhu et al., 2018; He et al., 2021)

### 3.1 Oral microbes in breast cancer

Female BC is actually the prevalent metastatic cancer and leading factor inducing cancer-associated mortality in females globally (Fatima et al., 2023). Based on the geographic variation and distribution of the disease, Arnold et al. have made a prediction that by the year 2040, the incidence of newly diagnosed BC cases will be elevated by over 40%, causing approximately 3 million patients every year, especially in transitioning countries. To address this growing burden, global initiatives and public health interventions that target the entire cancer control spectrum, including early diagnosis, primary prevention, screening, and treatment, are imperative (Arnold et al., 2022). Despite the wide attention and relatively comprehensive treatment options in clinical practice, BC still poses significant challenges in terms of understanding its pathogenesis and achieving precise prevention measures. Besides, 20% of BC cases belong to the TNBC category, characterized by a heightened propensity for recurrence, unfavorable prognosis, significant metastatic potential, and reduced survival rate (Lu et al., 2023).

The most common risk factors for BC include aging, family history, lifestyle factors, and endogenous/exogenous estrogen exposure (Niccolai et al., 2023; Sung et al., 2021). The human microbiota exerts significant influence on the occurrence and progression of BC, which may be the potential factor in early diagnosis. The complex microbial community residing in various body sites, including the breast tissue, makes a vital impact on keeping tissue homeostasis and immune regulation (Kim et al., 2021; Vitorino et al., 2022). Dysbiosis of microbial community is associated with inflammatory responses, genetic mutations, and changes in hormone metabolism that can promote carcinogenesis in the breast tissue (Niccolai et al., 2023; Saud Hussein et al., 2021). It was found that Bacteria isolated from breast tissue of Caucasian women exhibited the highest *Proteobacteria* and *Firmicutes* phyla relative abundances, alongside the presence of common oral bacteria including *Fusobacteria* and *Streptococcus* genera (Hao et al., 2019; Urbaniak et al., 2014). The oral microbiota is shown to have a potential effect on BC occurrence and development. *Proteobacteria* were found to be the predominant phylum within breast tissue. However, in the oral cavity, members of this phylum constitute a smaller portion of the overall bacterial community (Wade, 2013; Saud Hussein et al., 2021).

Significantly, *Fusobacterium nucleatum* (*F. nucleatum*) has been an anaerobic oral commensal that can be detected in BC tissue. Based on AXIS tool analysis, oral *Fusobacterium nucleatum* was positively related to BC. The high oral *Fusobacterium nucleatum* level, often associated with gingivitis/periodontitis, was found to increase the BC risk (Gaba et al., 2022). Based on Van der Merwe et al., *F. nucleatum* enhanced BC development via TLR4/MyD88 pathway activation, showing immunomodulation (Fan et al., 2023; Van Der Merwe et al., 2021). In recent studies, BC specimens exhibit the presence of *Fusobacterium nucleatum* chromosomal DNA, which is positively correlated with high levels of Gal-GalNAc and BC progresses. *Fusobacterium nucleatum* depends on its Fap2 to bind to BC specimens, which can be inhibited by GalNAc. Intravenous injection of *Fusobacterium nucleatum* expressing Fap2 can specifically colonize mouse mammary tumors. Injection of *Fusobacterium nucleatum* can hinder anti-tumor T lymphocyte accumulation and infiltration and enhance tumor development and migration, which is reversed through antibiotic application. Therefore, the study results suggest that colonization of *Fusobacterium nucleatum* in BC can accelerate tumor development and migration (Parhi et al., 2020). Targeting *Fusobacterium nucleatum* or its Fap2 during BC treatment may hold promise for therapeutic interventions. In another investigation about whether oral microbiome is related to BC and benign breast disease in Ghana, *Porphyromonas* and *Fusobacterium* have decreased relative abundances in BC patients relative to controls. Alpha-diversity and presence/relative abundance of specific genera in oral microbiome was closely related in BC patients (Wu et al., 2022c). This study suggests a potential correlation between the abundance of different bacterial taxa in cancer cases and geographical regions. In the Women’s Health Initiative Observational Study, a comprehensive and extensively studied prospective cohort of postmenopausal women, a history of diagnosed periodontal disease was associated with a higher susceptibility to primary, invasive BC. These findings are consistent with the notion that chronic inflammation may contribute to BC risk, and they indicate that oral microbiome may be involved in BC occurrence and prevention (Freudenheim et al., 2016).

Recent studies on BC patients receiving chemotherapy reveal dysbiosis of oral microbiota based on saliva data. Following chemotherapy, *Neisseria*, an aerobic genus found in the human oral cavity, has reduced abundance, while potentially pathogenic taxa including *Escherichia/Shigella* have significantly increased abundances. Our findings indicate that certain oral bacterial taxa are related to side effects of cancer treatment. Therefore, during chemotherapy, the oral microbial status during cancer treatment is of great importance (Klymiuk et al., 2022). However, there have been conflicting opinions concerning the possible association of periodontal disease with BC. Bernhard et al. suggested that there is no direct association between the subgingival microbiota including *Actinomyces gerencseriae* and BC by clinical and microbiological assessment in BC patients, and an indirect pathway should be addressed in further studies (Silva-Boghossian et al., 2018).

In another hospital-based study, it was observed that women diagnosed with periodontitis had a two to threefold increased likelihood of developing BC compared to women without periodontitis, and these findings strongly suggest that periodontitis is significantly related to BC (Sfreddo et al., 2017). In a recent prospective cohort study investigating the potential relation of periodontitis with invasive BC, no definitive association of periodontal disease with the overall risk of developing BC was found (Jia et al., 2020). Some scholars argue that there is no obvious evidence supporting the association of periodontitis with BC, the divergent outcomes across studies may be caused by variations in research design, assessment of periodontal health, confounder adjustment, duration of follow-up, and sample size (Issrani et al., 2021).

In conclusion, while the relation of oral microbiota with BC remains controversial, there is significant potential in exploring certain oral microbial species as risk factors for BC development and biomarkers. Investigating the intricate connection between oral microbiota and BC holds profound research value and promising prospects.

### 3.2 Gut microbes in breast cancer

The gut microbiota comprises a variety of microorganisms, including bacteria, yeast, and viruses. The major gut microbial phyla include *Firmicutes*, *Bacteroidetes*, *Actinobacteria*, *Proteobacteria*, *Fusobacteria*, and *Verrucomicrobia*. In the *Firmicutes* phylum, there are over 200 diverse genera, such as *Lactobacillus*, *Bacillus*, *Clostridium*, *Enterococcus*, and *Ruminicoccus*. *Bacteroidetes* mainly include *Bacteroides* and *Prevotella*. The *Actinobacteria* phylum has a relatively lower abundance, which is primarily represented by *Bifidobacterium* genus (Thursby and Juge, 2017; Rinninella et al., 2019). Gut microbiomes participate in the pathogenesis, produce inflammatory cytokines, and break the intestinal barrier integrity, promoting BC (Xia et al., 2023).

#### 3.2.1 Gut microbiota dysbiosis in breast cancer

As reported by Rosean et al., gut microbiota dysbiosis directly influences BC dissemination. Prior to tumorigenesis, a disruption of the gut microflora, can sufficiently promote collagen accumulation in tissue and tumor microenvironments, further resulting in systemic alterations corresponding to the more invasive HR+ BC (Buchta Rosean et al., 2019). In a clinical analysis on invasive cancer of non-specific type (HR+ and HER2-), results from newly diagnosed BC patients indicated that Shannon index, employed as a measure for diversity, significantly decreased in BC group relative to the control group. The study emphasizes that microbial diversity decreases, the *Firmicutes* relative abundance increases, and *Bacteroidetes* abundance reduces among early BC patients in comparison with normal controls. A trend to the reduced *Odoribacter sp*, *Butyricimonas sp*, and *Coprococcus sp* abundances could also be observed (Bobin-Dubigeon et al., 2021; Jaye et al., 2021). Based on this preliminary study, some variations may be observed in intestinal bacterial composition in BC patients compared with healthy individuals. Consistent with this conclusion, Haque et al. found the reduced gut microbiota diversity among BC patients, accompanied by the elevated *Clostridiales* abundance in relative to normal controls (Haque et al., 2022).

Recent studies have shown that *Bacillus*, *Staphylococcus*, *Enterobacteriaceae*, *Comamondaceae*, and *Bacteroidetes* exhibited significantly elevated relative abundances among BC patients (Saud Hussein et al., 2021; Urbaniak et al., 2016). A recent cross-sectional study first suggested that *Bifidobacterium* abundance in gut microbiota was positively related to omega-3 polyunsaturated fatty acids (PUFAs) in blood of BC survivors (Horigome et al., 2019). A retrospective analysis demonstrated that *Bacteroides* exhibit frequent and significant enrichment into the intestinal tract in BC patients (Wang et al., 2022). In another clinical study involving BC and Benign Breast Lesions (BBLs), BC patients exhibited increased levels of *Porphyromonas* and *Peptoniphilus* compared to healthy individuals, while the benign breast lesion group exhibited higher abundance of *Escherichia* and *Lactobacillus* in comparison (Ma et al., 2022). It is of note that specific gut microbiota abundance is related to difficult clinicopathological factors, including ER, PR, Ki-67, Her2, and hormone receptor+ or triple-negative levels (Nandi et al., 2023; Haque et al., 2022).

In investigations specifically addressing select groups of BC patients, Postmenopausal BC patients were shown to exhibit increased levels of *Escherichia coli*, *Enterococcus gallinarum*, *Actinomyces*, *Shewanella putrefaciens*, and *Erwinia amylovora*, while showing reduced levels of *Eubacterium eligens* and *Lactoisacus*. The intestinal metagenomes of postmenopausal BC patients exhibited the increased levels of genes related to lipopolysaccharide biosynthesis, a vital factor triggering systemic inflammation, which may potentially contribute to the promotion of neoplastic transformation (Barrea et al., 2023; Zhu et al., 2018). It was reported that two SCFA (short-chain fatty acids)-producing bacteria had substantially decreased abundances in premenopausal BC group, implying that *Pediococcus* and *Desulfovibrio* might be the diagnostic biomarkers for premenopausal BC (He et al., 2021). Some vivo studies showed that tumor-bearing mice exhibited compromised colonic barrier integrity, including decreased *Lactobacillus* abundance, and increased *Bacteroides* abundance in the orthotopic and syngeneic mammary tumor model. Various bacteria separated from spleens in these mice were determined to have originated from intestines (Loman et al., 2022). Intestinal bacteria including *Lachnospiraceae*, *Lactobacillus* and *Alistipes* were significantly changed in the xenograft nude mice challenged with BC cells (Zhang et al., 2021). Noteworthily, fecal microbiome in BC patients also displayed significant changes. According to Ma et al., *Faecalibacterium* had decreased abundance among BC patients, which was negatively associated with phosphorocholine. It indicates that the combination of detecting flora bacteria like Faecalibacterium and flora metabolites including phosphorolcholine, is beneficial for BC screening (Mikó et al., 2018; Ruo et al., 2021; Ma et al., 2020).

Collectively, these studies provide compelling evidence supporting that BC is related to obvious changes of gut microbiota. In addition, gut microbiome significantly affects the host overall health and physiology, establishing itself as a critical frontier in understanding BC pathogenesis.

#### 3.2.2 Metabolites and mechanisms associated with gut microbiota dysbiosis in BC

Gut microbiota includes diverse commensal microorganisms, including trillions of bacteria, viruses, and fungi. These microbial populations coexist in symbiosis with host; besides, their associated metabolites significantly influence human health. The gut microbiota makes an important effect on regulating metabolic, immune, and endocrine functions (Portincasa et al., 2022). Actually, current evidence strongly supports that metabolic disorders are crucial for microbial dysbiosis, therefore indirectly influencing the initiation and progression of neoplasms. Some of the key mechanisms include indirect processes like metabolite generation, including short-chain fatty acids (SCFAs). The glucuronidase activity of estroboloma bacteria, related to estrogen metabolism, also exerts a vital effect on this progression (Cazzaniga et al., 2022). Among the bacterial metabolites, short-chain fatty acids (SCFAs) such as acetate (C2), propionate (C3), and butyrate (C4), have the highest abundances in human body and colon, serving as prominent anions in the colon (Portincasa et al., 2022).SCFAs, secondary bile acids, and other metabolites are related to BC development, which may also be vital for modulating the response to chemotherapy (Plaza-Diaz and Álvarez-Mercado, 2023). SCFAs are a vital group of metabolites generated by bacterial species *Eubacterium rectale*, *Clostridium leptum*, and *Faecalibacterium prausitzii*, and by lactate-utilizing species *Anaerostipes* and *Eubacterium hallii*, directly influence can be exerted on different immune cells in the intestinal environment. Obviously, a substantial reduction in colonic SCFA levels has been found in premenopausal BC patients, indicating that SCFAs are vital for underlying pathological mechanisms of premenopausal BC (Zhang et al., 2022; Hill et al., 2014; Helmink et al., 2019). By being engaged with FFAR3, SCFAs impede the activation of MAPK signaling in BC cells, suppressing Hippo/YAP pathway, up-regulating E-cadherin, and prompting a transition of these malignant cells towards a non-invasive phenotype (Thirunavukkarasan et al., 2017; Yang et al., 2023). In the mouse model following BC cell injection, Zhang et al. reported that butyrate-producing genera, Lachnospiraceae and Ruminococcaceae, had decreased levels, which are known to promote the production of butyric acid and exert a protective role against colon cancer and triple-negative BC (TNBC) (Zhang et al., 2020a). Butyrate plays a crucial role as a nutrient source for colonocytes lining mammalian colon, and it is the histone deacetylase (HDAC) inhibitor. Sodium butyrate can stimulate apoptosis, induce ultrastructural changes, and enhance anti-tumor activity.

Besides, along with other HDAC inhibitors, Sodium butyrate has exhibited therapeutic effects on TNBC, particularly when used in combination with other anticancer agents (Zhang et al., 2021; Lee et al., 2016; Salimi et al., 2017; Wang et al., 2016; Sharma and Tollefsbol, 2022). Thus, the observed reduction in butyrate-producing genera, specifically *Lachnospiraceae* and *Ruminococcaceae*, in tumor-bearing mice supports the hypothesis that intestinal dysbiosis plays a role in enhancing BC. The following studies demonstrated that the gut microbiota-produced compound cadaverine exerts tumor-suppressive effects on BC by reversing epithelial-mesenchymal transition (EMT) and diminishing cancer cell stemness, motility, and metastatic characteristics (Kovács et al., 2019; Vergara et al., 2019). In addition, an *in-vitro* study findings revealed that the metabolite deoxycholic acid (DCA), specifically associated with *Clostridium*, enhances HER2-positive BC cell growth. It also stimulates G0/G1 phase cells transition into the S phase, potentially by activating peptide-O-fucosyltransferase and neuroactive ligand-receptor interaction pathway (Wang et al., 2022). Based on intestinal microbiota analysis, Vitamin D receptor (VDR) deficiency results in the shift of bacterial profile from normal to tumorigenesis risk in BC (Zhang et al., 2023d).

**Figure 1:**
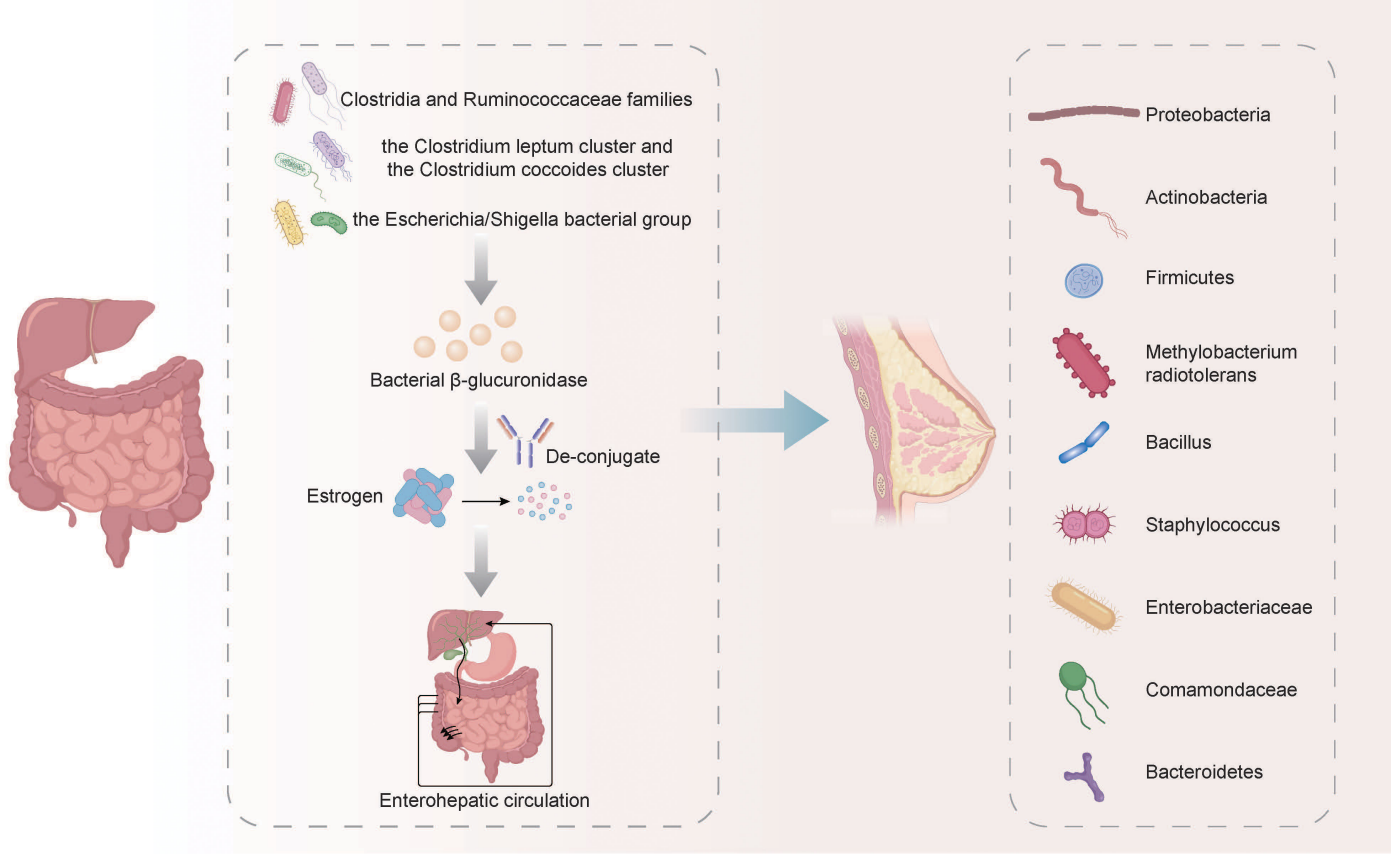
The metabolism of estrogens and its relationship with the microbiota in BC. The metabolism of estrogens occurs primarily in the liver, where they undergo conjugation and are subsequently excreted into the gastrointestinal tract through the bile. In the gut, these conjugated estrogens are deconjugated by bacterial β-glucuronidase enzymes produced by various bacteria, including members of the *Clostridia* and *Ruminococcaceae* families, the *Clostridium leptum* cluster, the *coccoides* cluster, and the *Escherichia/Shigella* bacterial group. After being deconjugated, the estrogens are then re-absorbed in their free form through the enterohepatic circulation, ultimately reaching the breast. The breast cancer tumors exhibit relatively high abundance of *Proteobacteria*, *Actinobacteria*, *Firmicutes*, *Methylobacterium radiotolerans*, *Bacillus*, *Staphylococcus*, *Enterobacteriaceae*, *Comamondaceae* and *Bacteroidetes*.

Sex hormone levels were closely related to some identified or suspected risk factors related to BC, and disruptions in microbiota/estrobolome may lead to increased levels of circulating estrogens and the corresponding metabolites, increasing the susceptibility to BC (Endogenous et al., 2011). The metabolism of estrogens occurs primarily in the liver, where they undergo conjugation and are subsequently excreted into the gastrointestinal tract through the bile. In the gut, these conjugated estrogens are deconjugated by bacterial β-glucuronidase enzymes produced by various bacteria, including members of the *Clostridia* and *Ruminococcaceae* families, the *Clostridium leptum* cluster, the *coccoides* cluster, and the *Escherichia/Shigella* bacterial group. After being deconjugated, the estrogens are then re-absorbed in their free form through the enterohepatic circulation, ultimately reaching the breast (Fernández et al., 2018; Rea et al., 2018). According to some relevant studies, the most common phyla in breast cancer tumors are *Proteobacteria*, *Actinobacteria*, and *Firmicutes*. Additionally, *Methylobacterium radiotolerans* are relatively abundant, and breast cancer patients exhibit significantly higher relative abundances of *Bacillus*, *Staphylococcus*, *Enterobacteriaceae*, *Comamondaceae*, and *Bacteroidetes* in their tumor tissues (Fernández et al., 2018; Xuan et al., 2014) (**Figure 1**). In the gut, microbes decompose indigestible dietary polyphenols into estrogen-like metabolites with biological activities which are generated through oxidation and reduction, and the induced estrogen-inducible growth factor production, such as estromedin by these bacteria may be potentially used for carcinogenesis (Alvarez-Mercado et al., 2023; Dabek et al., 2008; Toumazi et al., 2021). Certain commensal organisms possess the ability, through sulfatase activity, to convert inactive steroids into active estrogen. Stimulating estrogen receptors in the breast can promote cells that enter G0 and G1 phases, triggering BC cell growth. Activating mitochondrial estrogen receptors can promote mitochondrial oxidation, which leads to elevated production of free radicals, thereby promoting BC occurrence (Ruo et al., 2021). The potential therapeutic strategy of recovering gut microbiota balance and decreasing systemic estrogen content is recognized to lower the overall risk and advancement of BC. More efforts must prioritize the examination of gut microbial metabolites and the potential association with an increased BC risk.

**Figure 2:**
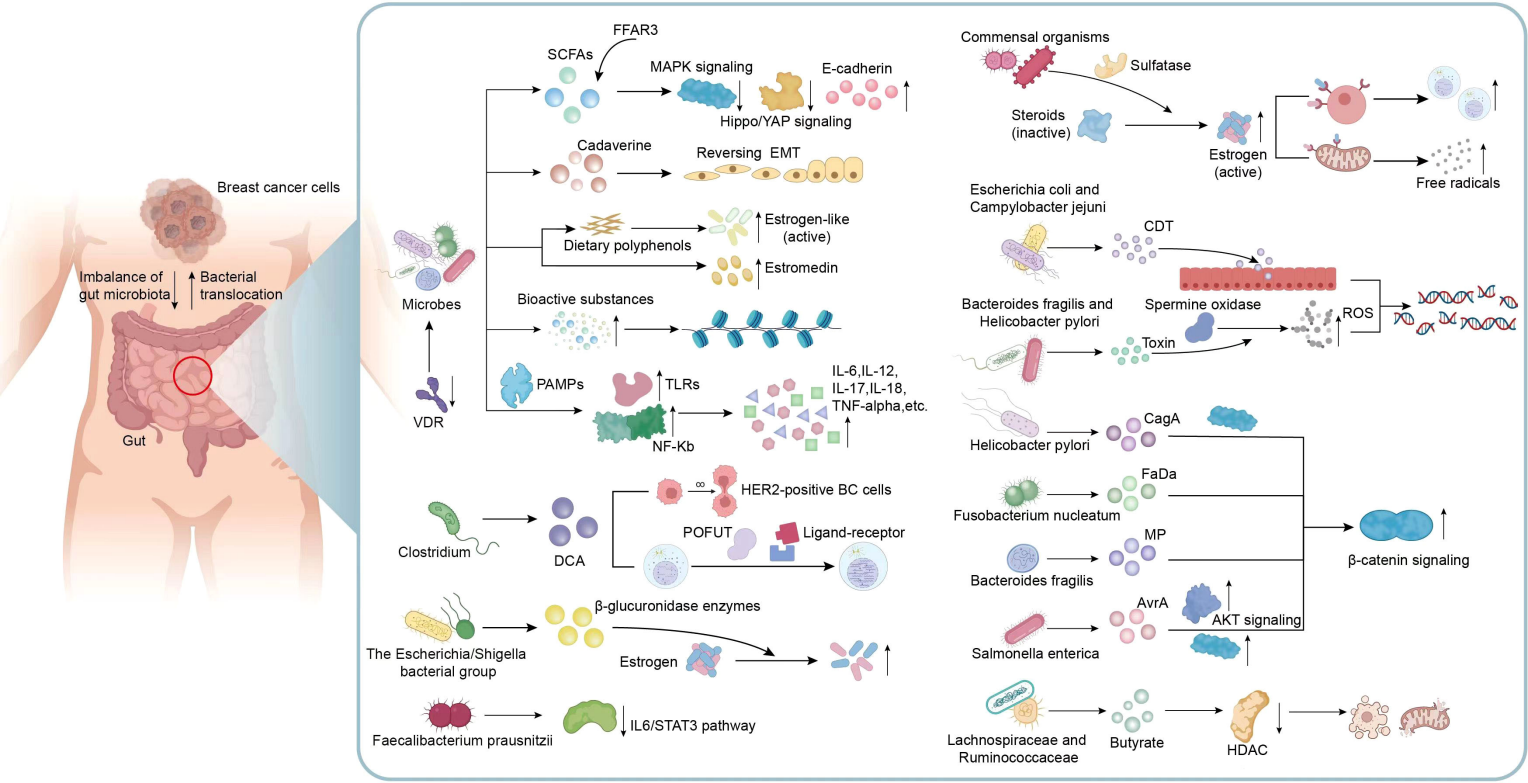
Mechanisms related to gut microbiota and metabolic dysbiosis in BC. Metabolic disorders exert a vital role in microbial dysbiosis, therefore indirectly affecting the initiation and progression of BC. Many microorganisms and their metabolites including VDR, SCFAs, Cadaverine and estrogen-like metabolites are involved in the mechanisms associated with the occurrence and development of BC, including the transition of invasive phenotypes, epithelial-mesenchymal transition, motility and metastatic characteristics, epigenetic processes, active estrogen production and inflammatory responses in the microenvironment. In addition, metabolites produced by certain specific bacteria are also involved in many signaling pathways associated with BC. For example, DCA specifically associated with *Clostridium*, enhances the proliferation of HER2-positive BC cells and regulate cell cycle; *Faecalibacterium prausnitzii* exhibited a suppressive effect on the growth of BC cells through inhibiting the IL6/STAT3 pathway; The toxins produced by *Bacteroides fragilis*, *Helicobacter pylori*, *E. coli* and *Campylobacter jejuni* can activate DNA damage and result in the formation of tumors; Metabolites from *Helicobacter pylori*, *Fusobacterium nucleatum*, *Bacteroides fragilis*, and *Salmonella enterica* possess the ability to activate β-catenin signaling, contributing to cancer; A reduction in the abundance of butyrate produced by *Lachnospiraceae* and *Ruminococcaceae* can attenuate anti-tumor activity. VDR, Vitamin D receptor; SCFAs, short-chain fatty acids; EMT, epithelial-mesenchymal transition; TLRs, Toll-like receptors; PAMPs, pathogen-associated molecular patterns; DCA, Metabolite deoxycholic acid; POFUT, peptide-O-fucosyltransferase; ROS, reactive oxygen species; CDT, cytolethal distending toxin; AKT, Protein kinase B, PKB; HDAC, histone deacetylase.

Obviously, dysbiosis of gut microbiota and disturbances in their metabolites can directly or indirectly impact cancer genesis and progression. Many microorganisms and the metabolites are related to signaling pathways linked to BC genesis and progression, as well as changes of surrounding microenvironment. Understanding these signaling pathways can shed novel lights on clinical interventions for the treatment of BC (**Figure 2**). Microbial composition in BC females differs from normal controls, in terms of bacterial species, their relative abundance, and functional attributes like metabolism and ability to induce DNA damage (Plaza-Díaz et al., 2019). Ana Isabel et al. proposed that double-strand breaks which pose the greatest threat among various DNA damage types resulting from genotoxins, ionizing radiation, reactive oxygen species (ROS), are probably related to BC development. Breast cancer is usually caused by epithelial cells and human exposure to carcinogens through diet and the environment encompasses contact with many fat-soluble genotoxins like polycyclic aromatic hydrocarbons (PAHs), nitro-PAHs, and heterocyclic aromatic amines which have the potential to trigger mammary tumors in rodents (Martin, 2001).Toxins generated by *Helicobacter pylori* and *Bacteroides fragilis* can activate the human enzyme spermine oxidase, which causes the production of ROS and hydrogen peroxide to result in DNA damage (Vivarelli et al., 2019; Alvarez-Mercado et al., 2023). When *E. coli* and *Campylobacter jejuni* can release cytolethal distending toxin (CDT) near the gastrointestinal epithelium, resulting in the generation of DNA double-strand breaks in epithelial cells. This process leads to a temporary cell cycle arrest and creates an environment in which mutations can occur, potentially leading to the formation of tumors (Alvarez-Mercado et al., 2023; Gori et al., 2019). In a study performed from Urbaniak et al., *Staphylococcus epidermidis*, separated in BC patients, could induce DNA double-stranded breaks. Noteworthily, other Enterobacteriaceae family members are also capable of producing colibactin (Urbaniak et al., 2016; Scott et al., 2019; Alvarez-Mercado et al., 2023). Gut microbiome also plays a role in disrupting epigenetic regulation, which can interact with the tumor. Microorganisms in the gut can generate low-molecular-weight bioactive substances including folates, SCFAs, and biotin. These substances are actively related to various epigenetic processes, such as modifying substrates for methylation or synthesizing complexes for modifying epigenetic enzyme activities (Vitorino et al., 2021; Haque et al., 2022).

The promotion of BC by gut bacteria occurs through inducing chronic inflammation, and it is associated with tumor formation. Gut bacteria stimulate upregulation of Toll-like receptors (TLRs) and activation of NF-kB through pathogen-associated molecular patterns (PAMPs). NF-kB activation triggers secretion of various factors such as IL-6, IL-12, IL-17, IL-18, and tumor necrosis factor-alpha (TNF-alpha), causing sustained inflammation within tumor microenvironment (Vitorino et al., 2021). In previous studies, The *H. pylori* cagA gene product, engages in a physical interaction with E-cadherin, disrupts the complex formation between E-cadherin and beta-catenin, leading to the accumulation of beta-catenin in the cytoplasm and nucleus regardless of CagA’s tyrosine phosphorylation status (Murata-Kamiya et al., 2007). *Fusobacterium nucleatum* (*Fn*) attaches to E-cadherin, targeting a specific 11-amino-acid region, thereby activating β-catenin signaling and exerting differential regulation on inflammatory and oncogenic responses (Rubinstein et al., 2013). In addition, metalloproteinase (MP) toxin in *Bacteroides fragilis*, and *Salmonella enterica*’s virulence factor A (AvrA) possess the ability to activate β-catenin signaling, further modulate cell growth, and survival pathways, therefore facilitating cancer development (Alvarez-Mercado et al., 2023). CagA protein derived from *Helicobacter pylori* exerts its influence on the MAPK pathway, while the AvrA factor produced by *Salmonella enterica* promotes the MAPK and AKT pathways (Alvarez-Mercado et al., 2023; Bronte-Tinkew et al., 2009; Vivarelli et al., 2019). It was clearly revealed that *Faecalibacterium prausnitzii* exhibited a suppressive impact on BC cell proliferation through suppressing IL6/STAT3 pathway. The above result suggests a potential avenue for BC treatment (Ruo et al., 2021).

Furthermore, the gut microbiota profile could be significantly altered by diet, obesity, alcohol consumption, and changes in circulating hormonal levels, indicating that microbiota make-up is strongly associated with BC occurrence. The disrupted microbiota, leading to changed microbial-related activities, probably influences the risk of developing BC (Smith et al., 2021; Bouter et al., 2017; Teng et al., 2021). The high-fat diet and presence of obesity affect the gastrointestinal microorganism composition and contribute to increased estrogen levels (Toumazi et al., 2021). The gut microbiota that produces the enzyme β-glucuronidase (GUS) has the potential to enhance estrogen bioavailability by breaking down estrogen-glucuronide conjugates, thereby facilitating their reabsorption into the circulation. Consequently, this increase in circulating estrogens may contribute to the development of estrogen receptor-positive breast cancer (BC) (Arnone and Cook, 2022). Therefore, these factors may serve as risk factors for BC occurrence (Feng et al., 2021). It is widely suggested that BC is related to gut microbiome. However, future investigations are required to explore the detailed mechanisms of various clinical observations mentioned. These findings provide novel clues for potential intervention targets that could be used in clinical treatment for BC.

### 3.3 The oral-gut axis in breast cancer therapies

BC exhibits significant variability in its clinical progression. Different treatments including chemotherapy and immune checkpoint inhibitors (ICIs) have emerged as promising therapeutic approaches. However, patients’ responses vary due to the heterogeneity of individuals, distinct characteristics of their BC, and the tumor microenvironment. The predictive factors for anti-BC immunotherapeutic response are still insufficient (Vitorino et al., 2021). Recently, BC and anti-BC treatment has shown bidirectional interaction with gut microbiota. For examples, accumulating evidence suggests that the gut microbiota modulates hormone therapy efficacy in HR+BC. Firstly, gut microbiota influences immune tone, therefore impacting the antitumor immunity triggered by hormone therapy.

Secondly, gut bacteria are capable of synthesizing, recycling, or degrading estrogens, thus influencing cancer cell-supportive hormone concentrations. Specifically, an overabundance of *Veillonella* genus bacteria has been observed in women with endocrine-resistant HR+ BC (Terrisse et al., 2023; Lasagna et al., 2022). 16S rRNA sequencing indicated that acupuncture treatment in CRF mice after BC chemotherapy increased Candidatus Arthromitus, *Lactobacillus*, and *Clostridia*_UCG-014_unclassified abundances, and reduced *Escherichia-Shigella*, *Burkholderia-Caballeronia-Paraburkholderia*, and *Streptococcus* abundances. Therefore, gut microbiota-gut-brain axis is impacted by BC chemotherapy (Lv et al., 2022). Chemotherapy is vital for regulating fecal metabolomic profiles in BC cases. Amino acids show up-regulation, while lactate and fumaric acid exhibit down-regulation among patients in second and third cycles compared with those prior to treatment. SCFAs displayed significant differentiation among those studied groups (Zidi et al., 2021). Mice harboring special microbial species, including *Akkermansia muciniphila*, *Bifidobacterium longum*, *Collinsella aerofaciens*, and *Faecalibacterium prausnitzii*, exhibited a more favorable response to anti-PD-(L)1 therapy (Vitorino et al., 2021). Commensal microbiota with abundance of *B. longum*, *Collinsella aerofaciens*, and *E. faecium* was related to anti-PD-1 therapeutic effect on cancers (Matson et al., 2018; Alpuim Costa et al., 2021). A direct correlation is found between the levels of *A. muciniphila* in fecal samples and the efficacy of anti-PD-1 therapy. Therefore, pharmacomicrobiomics research may promote using gut microbiota analysis for predicting ICI response in patients, contributing to the personalized and precision medicine for cancers (Vitorino et al., 2021).

Gut microbiome may influence tumor growth and the effectiveness of cancer treatment, making it a viable target for both tumor prevention and treatment strategies (Zhang et al., 2022; Muhammad et al., 2024). Currently, fecal microbiota transplantation is the primary approach for intestinal microbial therapy. Another method to restore the balance of intestinal microecological environment is using probiotics that holds significant promise as an antitumor strategy, including BC (Eslami et al., 2019; Thu et al., 2023; Zhang et al., 2023d). The improved therapeutic effect in mice receiving fecal microbiota transplantation (FMT) with responder feces was associated with the augmented CD45+and CD8+T cell activation in intestinal region (Routy et al., 2018; Vitorino et al., 2021; Alpuim Costa et al., 2021). Several studies have indicated an increased risk of immune-mediated toxicity in patients harboring specific bacteria, such as *Bacteroidaceae*, *Barnesiellaceae*, and *Rikenellaceae* (Vitorino et al., 2021; Chaput et al., 2017). This suggested that intestinal microbiota metabolites may be used for the treatment of BC cells. Toshitaka Odamaki et al. conducted a preliminary investigation using a randomized controlled trial design. Participants consumed yogurt containing the probiotic strain *Bifidobacterium longum* BB536 (BB536Y group), while the control group was provided with regular milk. The group consuming probiotic yogurt showed significantly reduced enterotoxigenic *Bacteroides fragilis* (ETBF) abundance in gut microbiome. This suggests that probiotic yogurt may have the potential to eliminate ETBF from the microbial community, potentially lowering the risk of breast hyperplasia and BC progression to some extent (Odamaki et al., 2012). Zhang et al. confirmed that gut microbiota can be regulated by *Lacticaseibacillus rhamnosus* Probio-M9 (Probio-M9), efficiently inhibiting BC development in BC cell-transplanted mice (Zhang et al., 2022). In overweight BC patients, probiotic treatment can substantially change composition and diversity of the intestinal flora, particularly the phyla *Bacteroidetes* and *Firmicutes* (Feng et al., 2021; Pellegrini et al., 2020). It was reported that Probiotic bacteria might be the candidate anti-BC prevention strategy among menopausal women. Dietary fiber, the well-known prebiotic type, can be fermented by gut bacteria producing cancer-protective metabolites like butyrate. Currently, some intervention clinical trials are under way to study how new probiotics can affect breast and intestinal microbiota in cancer patients and life quality in BC patients (Ruo et al., 2021). For examples, herbal supplements including American ginseng generate cancer-protective metabolites and modulate gut microbial composition for preventing tumor genesis (Rea et al., 2018). Ganoderma lucidum spores also regulate gut microbiota and recover normobiosis (Su et al., 2018). Maternal GE use can effectively prevent obesity-related metabolic disorders and BC subsequently in life through the dynamic influence on the interaction of early-life gut microbiota with important microbial metabolite profiles and offspring epigenome (Chen et al., 2022a). Jessica R Lakritz et al. demonstrated that consuming fermentative microbes including *Lactobacillus reuteri* may inhibit mammary carcinogenesis in animal model through stimulating lymphocytes (Lakritz et al., 2014).

However, Alastair proposed that gut microbial disruption by antibiotics negatively influences BC development and outcomes. The loss of beneficial microbial species induced by antibiotics may accelerate tumor development by changing mast cell homing and/or function (Mckee et al., 2021). Preexisting dysbiosis triggered by antibiotics is the host-intrinsic regulatory factor for tumor cell invasion and tissue inflammation in BC (Zhang et al., 2021). As discovered by Shiao et al., gut bacteria depletion using the cocktail containing ampicillin, imipenem, cilastatin, and vancomycin prior to radio therapy accelerated tumor development and reduced the survival in tumor-bearing mice compared with RT alone (Shiao et al., 2021; Zhao et al., 2023). It is indicated that gut microbiota affects the radiotherapy effect, even though such effect is negative.

It is of note that microbiota manipulation is applicable for improving therapeutic outcomes of BC patients. LCA acted as intestinal bacterial derivatives, and decreased SREBP-1c, FASN and ACACA levels in BC cells, exhibiting anti-proliferative and pro-apoptotic effects (Luu et al., 2018). It can also induce apoptosis in MCF‐7 cells through the binding to receptor G protein‐coupled bile acid receptor 1 (GPBAR1) and prevent epithelial-to-mesenchymal transition in BC cells by suppressing vascular endothelial growth factor (VEGF) expression (Trah et al., 2020; Yang et al., 2023; Chattopadhyay et al., 2022; Mikó et al., 2019). S-equol, the specific enantiomeric configuration of soy isoflavone metabolite generated by the human intestinal microbiota, effectively inhibited MCF-7 BC cell growth in both time- and dose-dependent manners. Moreover, S-equol facilitated apoptosis in MCF-7 cells by upregulating miR-10a-5p expression and inhibiting the PI3K/AKT pathway (Zhang et al., 2019). Metabolites derived from the *Faecalibacterium* genus exert growth-inhibitory efficacy in BC cells through inhibiting IL-6/STAT3 signaling pathway (Ma et al., 2020; Yang et al., 2023). As a highly abundant SCFAs generated in gut microbiota, butyrate has presented with potential anticancer activity against BC. It can be generated naturally as the bacterial fermentation by-product of fermentable and non-digestible carbohydrates, like dietary fibers, in GIT. Particularly, low butyrate content can cause tumorigenesis in host, while high content can suppress tumorigenesis and tumor development (Jaye et al., 2022). *Faecalibacterium prausnitzii* can inhibit BC cell development by inhibiting IL6/STAT3 pathway; thus, more efforts are needed to develop the candidate intervention (Ma et al., 2020). In breast and intestinal environment, colonization of enterotoxigenic *Bacteroides fragilis* (ETBF) can secrete a toxin called *Bacteroides fragilis* toxin (BFT), which rapidly induces proliferation of mammary ductal tissues (usually presenting as precancerous lesions of BC). This suggests that enterotoxigenic *Bacteroides fragilis* (ETBF) in the gut can be another possible risk factor related to BC development (Parida et al., 2021). In fact, gut microbial species are suggested to translocate to breast tissue, and it is vital for maintaining breast health. Such translocation may occur through several pathways, such as nipple-oral contact through lactation (Jaye et al., 2022; Hieken et al., 2016). Interestingly, recent research has demonstrated significant changes in oral and gut bacterial community compositions in dogs impacted by canine mammary tumors. Specifically, there was a substantial increase in *Bacteroides*, serving as an important microbial biomarker for oral and gut bacterial communities. This suggests a potential migration of microbiota from the mouth to the intestine and finally reaching distant mammary tumor tissue. This study presents the novel microbiological concept for treating canine mammary tumors, also providing a theoretical basis for the study of human BC (Zheng et al., 2022).

**Figure 3:**
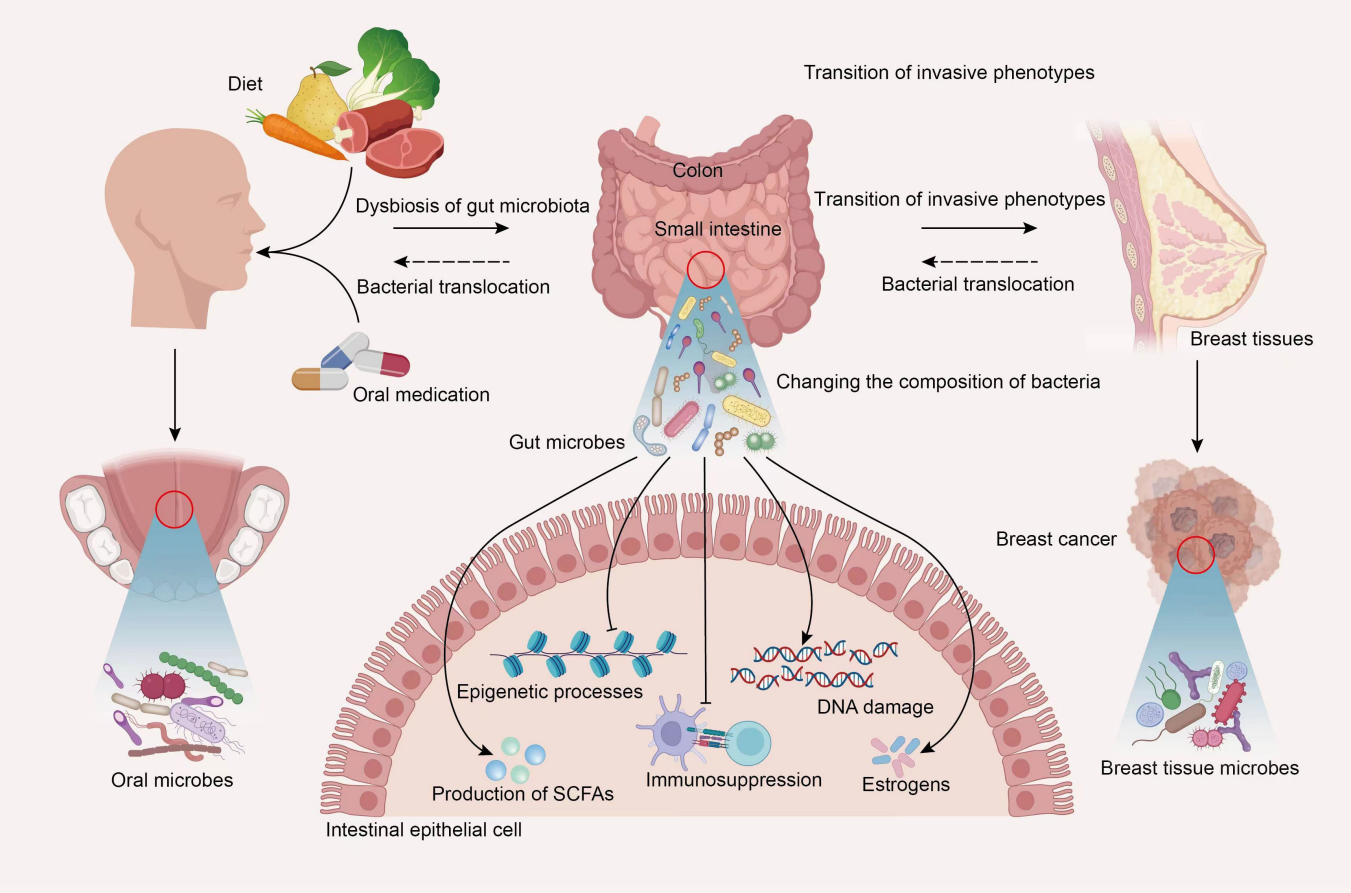
Hypothetical diagram of the interplay of microbiota within the oral-gut axis in BC. In specific situations, the oral microbiota profile could be significantly altered by diet, oral medication and more, certain external or oral microorganisms may overcome barriers between oral and gut environments and potentially migrate to intestines, thereby affecting the gut microbiota’s ecological balance. Gut microbiota and the metabolites may participate in the occurrence and progression of BC through various pathways, including influencing epithelial-mesenchymal transition, the transition of invasive phenotypes, DNA damage, epigenetic processes, inflammatory responses in the microenvironment, estrogen and short-chain fatty acids production, and more. This eventually reaches breast tumor tissues. The microbial ecosystem surrounding breast tumor tissues may also undergo certain changes. Gut microbes show direct or indirect effects on oral microbial composition by regulating host’s immune function. In addition, at different stages of BC occurrence, development, and clinical treatment, the cancer itself may also cause alterations of oral and gut microbial compositions.

Thus, more recent information will facilitate thinking of the correlation between the interactions of oral and gut microbiota and genesis, development, clinical treatment, and prognosis in BC. Similarly, BC may also influence the body’s microbiota, including the microbial population of the oral-gut axis. Oral cavity is the most common entry point for external substances in the body. Different foods, medications, and even breastfeeding during lactation period could affect normal distribution of microbial communities within oral cavity. In specific situations, certain external or oral microorganisms may overcome barriers between oral and gut environments and potentially migrate to intestines, thereby affecting the gut microbiota’s ecological balance. This leads to changes in the composition of gut bacterial communities. Gut microbiota and the metabolites may participate in the occurrence and progression of BC through various pathways, including influencing epithelial-mesenchymal transition, epigenetic processes, inflammatory responses in the microenvironment, estrogen production, and more. This eventually reaches breast tumor tissues. The microbial ecosystem surrounding breast tumor tissues may also undergo certain changes. Gut microbes show direct or indirect effects on oral microbial composition by regulating host’s immune function. In addition, at different stages of BC occurrence, development, and clinical treatment, the cancer itself may also cause alterations of oral and gut microbial compositions. Although research related to this topic still faces a lot of limitations, it is believed that more mechanisms will be elucidated in the future with technological advancements and increased attention to the association of oral-gut axis with microbiota in BC patients (**Figure 3**).

## 4 Conclusion and perspectives

In conclusion, changes of gut and oral microbiota may facilitate the BC pathogenesis and its therapeutic treatment options. In addition, oral administration of probiotics or therapeutic drugs may provide an alternative approach to modulating gut microbiota. Monitoring changes of gut and oral microbiota can provide new opportunities for predicting, understanding, and treating BC. Existing research suggests that although the interplay of gut-oral axis microbiota with BC occurrence and treatment is not as well-established as the association of gut microbiota with gastrointestinal cancers, tumor microecology is inherently a complex network where microbial shifts and interactions often occur unexpectedly. As a direction in tumor microecology research, the study of microbiota in the gut-oral axis still holds significant prospects and research opportunities.
